# A review on indoor environmental quality in sports facilities: Indoor air quality and ventilation during a pandemic

**DOI:** 10.1177/1420326X221145862

**Published:** 2022-12-21

**Authors:** Dadi Zhang, Marco A Ortiz, Philomena M Bluyssen

**Affiliations:** Chair Indoor Environment, Faculty of Architecture and the Built Environment, 2860 Delft University of Technology, Delf, The Netherlands

**Keywords:** sports facilities, indoor air quality, indoor environmental quality, ventilation, comfort, health

## Abstract

Because of COVID-19, the indoor environmental quality (IEQ) in sports facilities has been a concern to environmental health practitioners. To develop an overall understanding of the available guidelines and standards and studies performed on IEQ in sports facilities, an extensive literature study was conducted, with the aim of identifying: (1) indicators that are being used to assess IEQ in different sports facilities; (2) indicators that are potentially interesting to be used to assess indoor air, in particular; (3) gaps in knowledge to determine whether sports facilities are safe, healthy and comfortable for people to stay and perform their activities. The outcome indicates that most current standards and previous investigations on IEQ in sports facilities mainly focused on dose-related indicators (such as ventilation rate), while building-related indicators (such as ventilation regime) and occupant-related indicators (such as IEQ preferences) were rarely considered. Little attention is given to the fact that ventilation systems may play an important role in the air quality of the location, and few investigations have been performed on the transmission of SARS-CoV-2. This study recommends more research into both occupant and building-related indicators as well as cross-modal effects between various IEQ factors for developing future standards on sports facilities.

## Introduction

Due to COVID-19 (Coronavirus disease 2019) in the past two years, many public buildings, including most sports facilities, were closed for a period of time due to being under lockdown. While the initial response to the spread of the coronavirus was slow, the number of deaths was increased,^1^ as well as the amount of suffering from long covid.^2^ Clearly, the risk of becoming infected with COVID-19 is higher in crowded and inadequately ventilated spaces.^3^ What was already actively communicated by several researchers from the start of the pandemic,^4^ namely that airborne transmission is a route of transmission requiring serious consideration, was finally acknowledged by the World Health Organization (WHO) on 30 April 2021.^5^ COVID-19 has raised the awareness of the complexity of the challenges ahead in designing adequate ventilation to keep building occupants safe, healthy and comfortable.^6,7^ Considering the importance of sports activities for keeping a healthy lifestyle, the awareness of the significance of providing healthy and comfortable environments for playing sports, has increased. The question therefore arose, how can we ensure a healthy and comfortable indoor environment? On which basis can we determine whether these buildings are safe for people to stay and undertake sports activities both during a pandemic and during non-pandemic times? Specifically, what is needed to ensure a good indoor air quality (IAQ) in these sports facilities?

The quality of an indoor environment is determined by four indoor environmental quality (IEQ) factors: air quality, lighting quality, acoustical quality and thermal quality. These factors are interrelated: measures to improve IAQ might influence other IEQ factors.^8,9^ Which measures can be applied to assure a good IAQ without deteriorating the other factors, is therefore an important question to answer. The health and comfort indicators that are available to assess IEQ can be divided into three groups of indicators:^10^ (1) the dose-related indicators, using the dose or environmental parameters, such as concentrations of certain pollutants, temperature level or ventilation rate; (2) the occupant-related indicators, focussing on occupants such as sick leave, productivity and the number of symptoms or complaints; and (3) the building-related indicators, which are concerned with buildings and its components, such as certain measures or characteristics of a building and its components, or even labelling of buildings and its components. Of these groups of indicators, the first one (dose or environmental parameter related indicators) is used most frequently in standards and guidelines. Whether these standards and guidelines for IEQ in sport facilities can be used to assure health and comfort during pandemic and non-pandemic times, is questioned. Moreover, do current guidelines and standards for IEQ take account of different interactions and different requirements for different types of sports facilities (sports halls, swimming pools, fitness centres), different occupants (sporters, spectators and personnel), events (such as sports, concerts and educational activities) and activities (e.g. swimming, football, gymnastics, fitness, spinning, dancing and yoga)? If not, what is needed to fulfil these requirements?

To answer the questions above, an extensive literature study was performed, focussing on different standards and guidelines applied in different countries and worldwide research performed on IEQ in sports facilities, with a focus on IAQ. Because sports facilities comprise of a whole range of typologies, the choice was made to consider the three most common types in the Netherlands: Gymnasia and/or sports halls, pools, and fitness centres and studios.

## Methods

The literature study aimed at identifying:

1. Indicators used to assess IEQ in different sports facilities;
2. Indicators that are potentially interesting for use to assess IEQ, in particular IAQ, in different sports facilities;
3. Gaps in knowledge to determine whether sports facilities are safe, healthy and comfortable for people to stay and undertake their activities.

**Figure 1.**
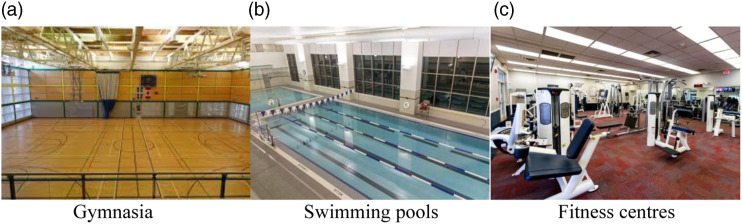
Different types of sports facilities. (a) Gymnasia (b) Swimming pools (c) Fitness centres.

Three different types of sports facilities (see Figure 1) covered in this study are:

- Gymnasia and/or sports halls^11^:
- Small Gymnasia used mainly by schools, usually are older buildings (before 1990)
- Sports halls, which usually include more buildings, are infrastructures dedicated to indoor team sports such as basketball, handball and volleyball, have restaurants, etc.
- Pools can be used for several aquatic sports such as swimming or water polo.^11^
- Fitness centres and smaller studios can offer a range of group classes and individual workout programs.^12^

**Figure 2.**
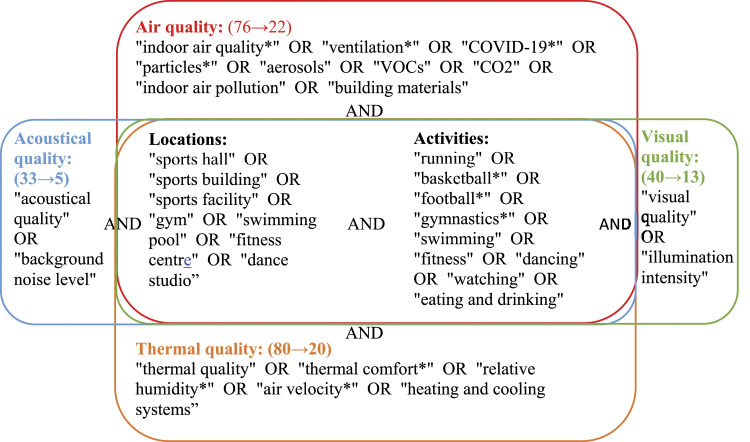
Keywords for literature search.

Related publications were collected by searching in Google Scholar, ScienceDirect, PubMed and Wiley. Conference proceedings of Indoor Air, Healthy Buildings, CLIMA and ASHRAE Annual Conferences were also included. Additionally, national and international standards, guidelines and requirements on IEQ in sports facilities were retrieved by browsing the official websites such as WHO, ISO, CEN and various national governments. The keywords used for the literature search are shown in Figure 2.

First, six queries (i.e. locations, activities, air quality, visual quality, thermal quality and acoustical quality) were created. Then, four separate searches were conducted for the four IEQ factors. Queries ‘locations’ and ‘activities’ were included in all searches. The relationships between queries are ‘AND’, while the relatio nships between the keywords within queries are ‘OR’. In total 229 publications were found based on the search. After careful selection, 60 were included in this review (detailed information can be found in Figure 2). Selection criteria included subject area (environmental science, engineering, social science), document type (article, conference paper, book chapter and review), language (English) and year (2000–2022). Additionally, 21 national and international IEQ-related standards and guidelines were checked.

## Results

### Current standards and guidelines on IEQ in sports facilities

Table 1 presents the current standards and guidelines on IEQ in sports facilities used in different countries. Some countries do not have specific standards on IEQ in sports facilities, or some of them are inaccessible. For the latter countries, the general standards on IEQ in all buildings, including sports facilities were reported.

**Table 1.** Standards and guidelines on IEQ in sports facilities used in different countries.

Region	Country	Standards/guidelines	Building type	IEQ-related indicators
World	World	WHO Guidelines for Indoor Air Quality: selected pollutants^13^	All buildings	Benzene, CO, formaldehyde, NO_2_, PAHs, radon, trichloroethylene, tetrachloroethylene
Asian	China	JGJ 31-2003 J 265-2003 Design code for sports buildings^14^	Sports hall/swimming pool	Temperature/relative humidity/air velocity/fresh air volume/reverberation time/illuminance
	Japan	Environmental Quality Standards in Japan^15^	All buildings including sports facilities	Concentrations of CO_2_/NO_2_/SO_2_/PM/benzene/trichloroethylene/tetrachloroethylene/dichloromethane
Europe	Europe	EN15251:2007 Indoor environmental input parameters for design and assessment of energy performance of buildings addressing indoor air quality, thermal environment, lighting and acoustics^16^	All buildings including sports facilities	Temperature/relative humidity/ventilation rate/CO_2_ concentrations/luminance/unified glare rating/sound pressure level
		EN 12193 Light and lighting – Sports lighting^17^	Sport halls	Luminance levels/uniformity of horizontal illuminance/colour temperature of the lighting
		EN 12464-1:2021 Light and lighting – Lighting of work places – Part 1: Indoor work places^18^	All buildings including sports facilities	luminance distribution/illuminance/glare/directionality of light/colour rendering and colour appearance of the light
		NEN-EN 16798 Energy performance of buildings – Ventilation for buildings^19^	All buildings including sports facilities	Ventilation rate/CO_2_/, temperature/relative humidity/reverberation time/horizontal illuminance/formaldehyde/glare angle
		1SO 16000-11 Indoor air – Part 11: Determination of the emission of volatile organic compounds from building products and furnishing – Sampling, storage of samples and preparation of test specimens^20^	All buildings including sports facilities	TVOC/SVOC/VVOC/aldehydes/
	The Netherlands	Handboek Huisvesting bewegingsonderwijs (2019)^21^	Sports hall	Temperature/air velocity/horizontal illuminance/evenness/background noise level. Reverberation time/ventilation rate
		Safety requirements for swimming pools – Design and management^22^	Swimming pool	Temperature/relative humidity/air velocity
		Dutch building decree^23^	All buildings including sports facilities	ventilation rate per person/ventilation rate per m^2^/inner surface temperature factor/air speed/dilution factor/lighting intensity/exposure time for undesirable noise
	UK	Code of Practice – The management and treatment of swimming pool water^24^	Swimming pool	Temperature/relative humidity/ventilation rate
	France	Public Health Code Sanitary rules (Code de la sante publique)^25^	Swimming pools	Transparency/the number of total coliforms/PH/germs concentration
	Spain	Regulation of thermal installations in buildings (RITE-2007) – IDA3^26^	All buildings including sports facilities	PMV/ventilation rate per person/CO_2_/olfactory comfort/a weighted noise level/
		NIDE 2021 – Halls and pavilions^27^	Sports halls and pavilions	Ventilation rate/CO_2_/, temperature/relative humidity/sound pressure level/illuminance
	Italy	CONI standards for sports facilities^28^	Sports hall/swimming pool	Illuminance/temperature/relative humidity/air change per hour/air velocity/background noise level/reverberation time
	Germany	DIN19643 – Treatment of water of swimming pools and baths^29^	Swimming pools	Water quality
		VDI 2089, Blatt 1^30^	Swimming pools	Relative and absolute humidity; ventilation
NorthAmerica	Canada	Residential indoor air quality guidelines^31^	Not specific for sports, based on residences	Various contaminants/PM_2.5_/VOCs
	USA	ASHRAE 62.1-2022 Ventilation and Acceptable Indoor Air Quality^32^	Sports hall/swimming pool/fitness centre	Ventilation rate
		ASHRAE Handbook – HVAC applications Chapter 6 – Indoor Swimming Pools^33^	Swimming Pools	Air change per hour/air velocity/relative humidity/air temperature
		ASHRAE Standard 55–2020 Thermal Environmental Conditions for Human Occupancy^34^	All buildings including sports facilities	Temperature/relative humidity/air speed

Note: CO = carbon monoxide; CO_2_ = carbon dioxide; NO_2_ = nitrogen dioxide; PAH = polycyclic aromatic hydrocarbons; PH = potential of hydrogen; PM = particulate matter; PM_2.5_ = particles smaller or equal than 2.5 micron; PMV = predicted mean vote; VOC = volatile organic compounds; SO_2_ = sulphur dioxide; TVOC = total VOC; SVOC = semi-VOC; VVOC = very VOC.

In terms of indicators used, they are quite consistent among these standards and guidelines and most of them are dose-related indicators. Additionally, there is no significant difference between these indicators and the indicators used in other types of buildings (such as offices or schools). For IAQ, the most used indicators in these standards and guidelines are ventilation rate and concentrations of indoor pollutants, such as formaldehyde and particulate matter (PM). For thermal quality, the indicators are temperature, relative humidity and air velocity. For visual quality, illuminance level and lighting uniformity are the most used indicators, while for acoustical quality, reverberation time and background noise are widely used.

ASHRAE has revised its IAQ-related standard ASHRAE 62.1 recently and changed the title from ‘Ventilation for Acceptable Indoor Air Quality’ to ‘Ventilation and Acceptable Indoor Air Quality’,^32^ which reflects ASHRAE’s recognition that IAQ goes beyond ventilation control. Although the minimum ventilation rate is still the main indicator used in this standard, information about certain contaminants and contaminant sources (including outdoor air, construction processes, moisture and biological growth) are also mentioned to guide the improvement of IAQ.

Apart from the dose-related indicators for IEQ, building-related indicators, such as occupancy, dimensions of a space, type of ventilation system, maintenance and cleaning schedules, type of materials used, type of lighting system and even colours of walls, floors and ceilings, can be important for keeping a healthy and comfortable environment in sports facilities. Some of these indicators may affect the IEQ directly.

Because these standards and guidelines for building-related indicators applied in different countries were difficult to assess or are not open to the public, this study mainly focused on the standards and guidelines used in the Netherlands. As mentioned in the Dutch Building decree,^23^ sports facilities are often multi-functional, such as holding exhibitions or examinations, therefore, besides the requirements for sports functions, they must meet the requirements for meeting and educational functions as well. Table 2 lists several requirements of the building-related indicators mentioned in the Dutch Building decree,^23^ the Dutch Handbook housing Physical Education,^21^ the NOCNSF-US1-RU.1^35^ and the Handbook for fitness centres^36^ that might affect the IEQ in sports facilities.

**Table 2.** Requirements of building-related indicators for sports facilities in Dutch standards and guidelines.

Standards/Guidelines	Articles	Indicators	Functions	Requirements
Dutch building decree^23^	1.2	Minimum number of persons per m^2^ of a staying area	Sports	0.05
			Watching sport	0.75
			Education	0.33
	4.7	Height of staying area	Sports	2.1 m
			Education	2.1 m
Handboek Huisvesting bewegingsonderwijs (2019)^21^	6.1	Floor area of the gym	School gym	252 m^2^
		Height of the gym	School gym	5 m
	2.3	Maximum group size	School gym	30 students +1 teacher
NOCNSF-US1-RU.1^35^	Normblad 3/4: Competition	Dimensions: minimum “free” height	Sport halls competition badminton, basketball, handball, volleyball, hockey	5.5 m 7 m
NL-Actief: Concept Handbook^36^	10.2.4	Flooring material	Fitness facilities	Cleanable, no source for moulds, non-hygroscopic (no carpet)
	10.2.4	Flooring walls ceiling	Fitness facilities	Colour reflection factor between: 0.25 and 0.40 0.45 and 0.60 0.60 and 0.80
	11.1	Ventilation	Fitness facilities	Quality and distribution of air are important

Besides the above-mentioned standards and guidelines, in several European countries labelling schemes are available for assessing whether a certain product stays below emission limits for certain VOCs, carcinogens and odours. For example, the AgBB protocol for flooring materials,^37^ which is a regulatory scheme, and the voluntary scheme M1 (Emission Classification of Building materials) of Finland.^38^ Most of these labelling schemes are based on the protocol described in ISO 16,000-Part 11.^20^

### IAQ in sports facilities

IAQ is an important factor to consider in sports facilities, however, no consensus exists as to which parameters are of greatest concern to occupants, whether trainers or spectators alike, as these parameters can be different from location to location. Some of the most prevalently monitored parameters in sports facilities, in different countries, are CO_2_, relative humidity, PM, certain VOCs and NO_2_. Additionally, ventilation requirements are frequently considered, such as air changes per hour, the maximum air velocity, and, less frequently, the ventilation per unity of surface and the perceived air quality in the facility.

A review of studies performed in three diverse types of sports facilities is presented: sports halls, swimming pools and fitness centres. Specifically, the review includes the indoor air quality requirements for sports halls, swimming pools and fitness centres, respectively, with a focus on the main problems found at each type of location and the methods used for assessment as well as the common indicators for assessment.

Table 3 presents the pollutants monitored in the indoor air quality studies that are reviewed in this paper. Six main pollutants are commonly investigated in sports halls (NO_x_, magnesia alba, acetone, CO_2_, formaldehyde and VOCs), four in fitness centres (PM_10_, VOCs, fungi and yeasts, and bacteria) and four in swimming pools (PM_2.5_, VOCs, fungi and yeasts, and water disinfection by-products), regardless of the ventilation system present at each type of location.

**Table 3.** Main pollutants, their sources, effects on humans, location and ventilation system, per type of sports facility presented in the reviewed studies.

Pollutant	Source	Ventilation type	Facility type	Effects on human health	Ref.
NO_x_	Outdoor traffic	Natural Mechanical	Sports halls	Cough Shortness of breath Tiredness Nausea	^39,40^
Magnesia alba	Occupant use	Natural Mechanical	Sports halls	Skin and respiratory irritant Cough Wheeze Chest tightness	^39,41,42^
Acetone	Polish Wax Detergents Cleaners	Natural	Sports halls	Eye and respiratory irritant Headache Dizziness Light headedness Nervous system effects	^39,40^
CO_2_^1^	Occupancy Athletes Spectators Personnel	Natural Mechanical	Sports halls	Tingling Dizziness (high exposure)	^43^
Formaldehyde	Composite wood Products with urea-formaldehyde resin Finishings Tobacco smoke Combustion processes Pressed wood adhesives	Natural	Sports halls	Eye, nose and throat irritation (low exposure) Rashes Shortness of breath Wheezing (high exposure)	^39^
PM_10_	Climbing chalk (magnesia alba) Resuspension from physical activity Cleaning activities	Natural Mechanical	Sports halls Fitness centres	Cough Asthma exacerbation Bronchitis	^41,44-46^
PM_2.5_	Resuspension from physical activities	Mechanical	Swimming pools	Eye, nose, throat, lung irritant Cough Sneeze Runny nose Shortness of breath	^47-49^
VOCs	Cleaning activities Disinfection by-products Alcohol-based disinfectant	Natural Mechanical (influenced by the efficiency of air filtration)	Sports halls Swimming pools Fitness centres	Eye, nose and throat irritation Shortness of breath Headaches Fatigue Nausea Dizziness Skin problems	^39,40,48-50^
Fungi and yeasts	High humidity Activities (perspiration, respiration)	Natural (fitness centres) Mechanical (swimming pools)	Swimming pools Fitness centres	Respiratory disorders Allergies	^51^
Bacteria	Air velocity Relative humidity High temperatures	Mechanical	Fitness centre	Respiratory disorders	^52^
Trichloramine Trihalomethanes Chloroform Bromoform Bromodichloromethane	Water disinfection with bleach	Mechanical ventilation (HVAC)	Swimming pools	Shortness of breath Eye, nose, throat irritation	^53-55^

^1^As an indicator of human bio-effluents.

Table 4 presents the requirements of dose-related ventilation indicators for assessing sports facilities presented in standards and guidelines of the Netherlands, CEN and ASHRAE. Similar as the information shown in Table 1, ventilation rate is the most used indicator for assessing IAQ.

**Table 4.** Requirements of dose-related IAQ indicators for sports facilities.

Standards/Guidelines	Articles	Indicators	Functions	Requirements
Dutch building decree^23^	Article 1.2 Number of persons	Capacity (persons/m^2^ of staying area)	Sports	0.05
			Watching sports	0.75
	Article 3.29 Ventilation in staying area, staying space, toilet space and bathroom space	Ventilation	Sports	0.7 dm^3^/s⋅m^2^ or 7 dm^3^/s
			Toilet	7 dm^3^/s
			Bathroom	14 dm^3^/s
	Article 3.33 Location of the opening	Dilution factor	sports	0.01
Handboek Huisvesting bewegingsonderwijs (2019)^21^	6.8 Ventilation	Ventilation rate	sports hall for education	40 m^3^/h/p 7 m^3^/h/m^2^
	8.1 Changing room and washroom	Ventilation rate (ACH)	showering	10 h^−1^
NOCNSF-US1-RU.1^35^	Normblad 3/4: Competition	Ventilation rate	sport halls: players spectator	40 m^3^/h p 20 m^3^/h p
NL-Actief: Handbook^36^	11.3	Ventilation rate (ACH = air changes per hour)	fitness facilities	6 h^−1^ (summer); 4 h^−1^ (winter)
			Fitness lessons	8 h^−1^ (summer); 6 h^−1^ (winter)
			spinning	10 h^−1^ (summer); 8 h^−1^ (winter)
			body & mind	6 h^−1^ (summer); 4 h^−1^ (winter)
EN15251:2007^16^	Table B.2	Ventilation rate	Auditorium	15 L/s/m^2^ (Ⅰ) 10.5 L/s/m^2^ (Ⅱ) 7 L/s/m^2^ (Ⅲ)
	Table B.4	CO_2_	All buildings including sports facilities	350 ppm (Ⅰ)^1^ 500 ppm (Ⅱ) 800 ppm (Ⅲ) >800 ppm (Ⅳ)
NEN-EN 16798 Energy performance of buildings – Ventilation for buildings^19^		CO_2_	All buildings including sports facilities	550 ppm (Ⅰ)^1^ 800 ppm (Ⅱ) 1350 ppm (Ⅲ)
ASHRAE 62.1-2022 Ventilation and Acceptable Indoor Air Quality^32^	Table 6-1	Ventilation rate	Gym, sports area	10 L/s/p; 0.9 L/s/m^2^
			Spectator area	3.8 L/s/p; 0.3 L/s/m^2^
			Swimming pool	2.4 L/s/m^2^
WHO Guidelines for Indoor Air Quality: selected pollutants^13^		Benzene	There is no known exposure threshold for the risks of benzene exposure. Therefore, from a practical standpoint, it is expedient to reduce indoor exposure levels as much as possible	
		CO		15 min: 100 mg/m^3^ 1 h: 35 mg/m^3^ 8 h: 10 mg/m^3^ 24 h: 7 mg/m^3^
		Formaldehyde		0.1 mg/m^3^ (30-min average concentration)
		Naphthalene		0.01 mg/m^3^ (annual average concentration)
		NO_2_		1 h average: 200 μg/m^3^ annual average: 40 μg/m^3^
		PAHs		0 (all levels are considered to be relevant to health
		Radon		Non-smoker: 0.6 × 10^−5^ Bq/m^3^; smoker (15–24 cigarettes per day): 15 × 10^−5^ Bq/m^3^
		Trichloroethylene		4.3 × 10^−7^ μg/m^3^
		Tetrachloroethylene		Annual average: 0.25 mg/m^3^

^1^recommended CO_2_ concentrations above outdoor concentration.

#### IAQ in sports halls (schools/public)

Several studies focus on the difference between indoor and outdoor air by monitoring NO_2_. Generally, NO_2_ is found to be higher outdoors, due to the presence of nearby traffic. Furthermore, TVOC tend to spike during the cleaning activities and decrease overnight. Acetone is another pollutant found to be higher in sports halls. The source of acetone has been assumed to be wood polishers and waxers, certain detergents, glues and cleansers.^39,40^

Another pollutant that is prevalent in sports halls is magnesia alba. Using this chemical, magnesium carbonate hydroxide is popular amongst athletes for drying their hands in different disciplines (climbing, bouldering, weight lifting, gymnastics, etc.).^41,42^ Magnesia alba is attributed as the main reason for the high particle levels of PM_10_ in sports halls.^39^ In most studies conducted in large sports halls, whether in schools, universities or public spaces, PM_10_ was found to be an important pollutant and seemed to be mainly dependent on the activities exercised in the halls, occupancy rates and cleaning activities. Additionally, there are specific activities that stimulate the resuspension of particles (from PM_2.5_ to PM_10_), namely those that make use of foam mats.^41,44,45^

Other researchers focused on the infection risk of diseases in sports halls with different ventilation systems (split-airco unit and central air conditioning). In one study the risk of influenza and tuberculosis was analyzed by assessing CO_2_ concentration levels and by using the Wells-Riley model. Results showed that regardless of the ventilation, the risk of infection was highest during the evenings, with the highest occupancy levels. The researchers suggested that sports halls are in need of efficient ventilation systems, that can maintain CO_2_ concentration at a low level to reduce the risk of viral infection.^41^

Suggestions to improve the IAQ in sports facilities include adequate mechanical ventilation with appropriate filters, suitable cleaning practices and limited occupancy. It is also suggested that because in these locations people increase their breathing rate, air quality requirements should be stricter.^56^

#### IAQ in swimming pools

Swimming pools can present a different challenge to the IAQ of a space, caused by the large volume of high humidity and temperature. Both personnel and athletes in swimming pools and aquatic centres can suffer from eye, nose and breathing symptoms, due to the chlorinated chemicals in the air released from the chlorination of water and exposure to disinfectants and their by-products. The exposure of such pollutants can occur through ingestion of the swimming water, inhalation of the compounds or by skin contact.^57^ Swimming pool chlorination can cause the emission of trichloramine and trihalomethanes, such as chloroform.^53,54^ PM_2.5_ concentrations in swimming pool halls tend to be higher than in other sports facilities, likely due to the chlorination used in the disinfection process of the water.^47^ Furthermore, chloroform is the main emission coming from pool water into the air; while other VOCs are negligible.

Other pollutants that have been found in swimming pool halls and aquatic centres are fungi and yeasts in suspension, in the air, water or on surfaces, and can represent a health risk to swimmers and personnel. These yeasts and fungi were of the species Penicillium, Aspergillus, Cladosporium and Alternaria, which were detected in the air and surfaces sampled in ten pools in Italy.^51^

Suggested solutions for the control of the emission of trichloramine and trihalomethanes to keep concentrations below 0.3 mg m^-^^3^ are to separate solid particles from the swimming water to form loose aggregations or soft flakes (flocculation), the use of adequate ventilation with appropriate filters, control of water flow and filtering, regulating water quality and ensuring good public hygiene to avoid urea, a dominant organic compound also found in swimming pools, present in sweat and urine.^53,55^ Similarly, one study found that the concentrations of urea tend to be higher with higher occupancy and usage of the swimming pools. VOCs emitted from disinfectant by-products also tended to be higher on Mondays and gradually decreased over the week, due to volatilization.^50^

Finally, in the latest studies regarding SARS-CoV-2 in swimming pools, the virus has been found susceptible to being inactivated in chlorinated swimming pool water. More specifically, its infectious concentration seems to reduce after 30 s. However, the inactivation in chlorinated swimming pool water is less effective as the water’s pH increases.^58^

#### IAQ in fitness centres

Fitness centres are a smaller type of sport facility than the aforementioned ones and might therefore present different risks to their occupants. Moisture resulting from the perspiration from physical activities may increase microbiological proliferation in fitness centres.^52^

In a study in which different fitness centres were compared based on their ventilation types (mechanical ventilation, natural ventilation and outdoor centre), the relative humidity, PM_10_, bacteria and fungi were found to be statistically different. More specifically, fitness centres with mechanical ventilation or air conditioning systems showed increased concentrations of PM_10_ levels.^46^ In another study mechanical ventilation was compared with natural ventilation in terms of bacterial concentrations in fitness centres. The results showed that naturally ventilated fitness centres can have higher concentrations of bacteria than mechanically ventilated ones. Also, mechanically ventilated centres can have higher bacterial concentrations than outdoors, due to the occupants’ activities. Fungi levels were, however, lower in mechanically ventilated centres, while naturally ventilated ones had much higher levels.^52^ Moreover, in a study on 11 fitness centres in Lisbon,^48^ regardless of the ventilation (mechanical, hybrid, natural), high levels of CO_2_ were found, indicating insufficient ventilation. The location of air intakes, in the case of mechanical ventilation, seemed to play a key role in the CO concentrations found (traffic being the source). Furthermore, VOCs spiked beyond limit values during the cleaning activities.^48^ Additionally, PM_2.5_ concentration levels tended to increase as the occupants increased their physical activity level indoors.^48,49^

SARS-CoV-2 was also investigated in fitness centres in Oslo, Norway, in a randomized controlled trial. Although the study did not report on the type of ventilation system present in the targeted fitness centres, the authors suggest that good hygiene from occupants, along with physical distancing and low occupancy, can reduce the risk for infection in fitness centres.^59^

### Other IEQ factors in sports facilities

Apart from air quality, other IEQ factors (i.e. thermal quality, acoustical quality and lighting quality) are also important to the occupants’ wellbeing in sports facilities, including but not limited to sports practitioners, spectators and employees. All the IEQ factors can affect occupants’ performance, health and comfort. Therefore, requirements are included in standards and guidelines to control these IEQ factors sports facilities.^21,33,60^ Also, for these IEQ factors in sports facilities several studies have been performed to determine the effect on health and comfort of the occupants.^61–63^ Several suggestions for improvement have been made.

#### Thermal quality in sports facilities

Standards and guidelines for thermal quality are different for different sports facilities. As shown in Table 5, the requirements for relative humidity and air temperature in swimming pool centres are relatively higher than for other sports facilities.^33^ While the requirements for air velocity in sports halls, especially during table tennis or badminton, are relatively lower to avoid the effect of airflow on the movement of a small object, such as a ping pong ball or a shuttlecock during play.^63–66^ In addition to these dose-related indicators, the adaptive thermal comfort model also plays an important role in determining thermal quality in an indoor environment.^67^ This model takes occupants-related indicators, such as clothing and metabolic rate into consideration. Based on this model, both the ASHRAE Standard 55^34^ and the ISO 7730 standard^68^ were adapted.

**Table 5.** Requirements of dose-related indicators for thermal, acoustical and lighting quality in sports facilities.

Indicators	Sports hall^1^	Swimming pool^2^	Fitness centres^5^
Thermal quality			
Air temperature	18–20°C^3^ ≤ 25°C	The difference with water temperature ≤ 4°C; ≤ 30°C^4^	High intensity: 15–18°C Normal intensity: 18–21°C light intensity: 20–25°C
Relative Humidity		40–80%; 60% recommended	< 60% recommended; < 70% requirement
Air velocity	≤ 0.2 m/s	≤ 0.5 m/s^4^	≤ 0.2 m/s
Acoustical quality			
Reverberation time	≤ 1.8 s^69^ ≤ 1.0–2.3 s^21^	1.5–2.0 s	0.6–2.3 s (depends on the volume of the space)
Background sound pressure level	40 dB(A)	—	40 dB(A)
Maximum sound pressure level		—	65 dB(A)
Average absorption coefficient (α)	≥0.25	—	—
Lighting quality			
Horizontal illuminance	500 lux	200 lux (on the water surface)	300 lux
Evenness	≥ 0.7	≥ 0.7	0.7
Daylight	≥ 5% of the floor surface	—	—
Colour temperature of lighting	—	—	3000 K or 4000 K
Colour rendering index R_a_	—	—	minimum 80

^1^Refers to Dutch Handbook Housing Physical Education^21^.

^2^Refers to Safety requirements for swimming pools design and management^60^.

^3^Desired operating temperature for educational use.

^4^Refers to ASHRAE handbook^33^.

^5^NL-Actief Handbook.^36^

Additionally, requirements for different areas in the same facility might be different.^70–72^ Usually, requirements for air velocity, air temperature and relative humidity in the playing area are stricter than those for other areas, because those parameters can influence athletes’ performance. For example, a low air temperature in a gym impaired women’s exercise performance,^73^ while a high air temperature was found to decrease football players’ performance.^61^ Then, studies have shown that air temperature affects the attention of teenagers more than that of adults,^74^ which resulted in a quite narrow temperature range, 18–20°C, recommended for physical education in sports halls.

To fulfil different thermal requirements in different zones in the same sports facility for different thermal demands of occupants, areas should be divided based on different requirements and have possibilities to control the temperature separately.^70^ Computational fluid dynamics (CFD)-simulations have been used to predict the air distribution in sports facilities during the design process to improve thermal comfort.^70,75,76^ In addition, considering the different thermal demands caused by different sports activities and levels among different people in the same sports building, locally controlled or personally controlled thermal systems, such as personal ventilation, have been suggested.^77^ An additional advantage of localized airflow or personal ventilation is the potential for saving energy.^77,78^

Next to the impact on performance and comfort, indoor thermal quality may also affect the spread of COVID-19.^79,80^ In a large-scale study involving almost 70,000 cases in China and more than 700,000 cases in USA, significant negative relationships were found between temperature/relative humidity and the effective reproductive number (which is an index for transmissibility). This means that a high temperature and a high relative humidity might have the potential to reduce the transmission of SARS-CoV-2 (spread of COVID-19).^81^ A similar relationship between temperature and transmissibility was found by Oliveiros et al.,^82^ however, in terms of the effect of humidity, the result was the opposite. They concluded that a high temperature could delay the spread of COVID-19, while a high humidity could benefit it. In both studies,^81,82^ the impact of thermal conditions on the transmission of SARS-CoV-2 was small, and lots of other factors could also have significant impacts. In recent studies performed by Morris et al.,^83^ SARS-CoV-2 survived longest at low temperatures and extreme relative humidities, both low and high.

#### Acoustical quality in sports facilities

Acoustical quality in sports facilities is also very important, and a great challenge for acoustical experts to improve because the noise type (airborne or structure-borne) and frequency can vary a lot depending on the sports activity.^84^

Reverberation time (RT) is one of the major parameters used to evaluate acoustical performance in buildings. In the Netherlands, 1.8s has been seen as the threshold of a good acoustical environment in sports halls.^69^ However, according to Nijs and Schuur,^69^ this is not a hard rule. The optimal value of RT depends on the volume of the hall. For big halls, an RT between 2.0 and 2.5 s is still acceptable, while for small halls, this value might drop to 1.5 s. They, therefore, suggested to use the mean value of absorption coefficient s(α) as the parameter to assess the acoustical quality of a sports hall.^69^ Likewise, in the Dutch guidelines for gymnasiums and sports halls for educational school use,^20^ no fixed value for RT is given. Instead, a minimum average absorption coefficient of a sports area is set at 0.25, and a list of maximum RTs is given for different sizes of the sports hall.

In primary schools, acoustical problems have been identified as the biggest problems,^85^ similar to findings in school gyms.^62^ In a series of field studies conducted by Carvalho and Barreira^62^ at 68 Portuguese high school gymnasiums, almost no sound-absorbing materials were present, the measured RTs were quite high (median 4.8s), and speech intelligibilities monitored were low. These gymnasiums failed to provide a healthy and comfortable environment for both students and teachers. The sound levels (median 80 dB) they were exposed to might even be harmful to them. Most teachers reported to be strongly bothered by the noise during the classes. To protect the health of these physical education teachers, the Dutch Royal Association for Physical Education (KVLO) recommended separating the school sports hall into three parts, while no more than two parts can be used at the same time.^21^

To improve the general acoustical quality in sports facilities, the construction of the floor (e.g. floating floor) as well as the flooring material applied, have been considered.^84,85^ Masoumi et al.^84^ conducted a set of drop weight tests to investigate the acoustical performance of different floors. They found that the heavy concrete floor was better performing at low frequencies, but the damped lightweight floor performed the best in general. However, the selection of flooring materials also depends on the function of the facilities. For example, for a basketball hall, hardwood is the most used flooring material, while for fitness or aerobic rooms, a resilient flooring material, such as rubber or PVC (polyvinyl chloride), is more common.^85^

#### Lighting quality in sports facilities

The lighting system in sports facilities should be able to provide a good visual quality for sports practitioners, referees and spectators. Appropriate visual conditions, which include proper illuminance levels and uniformly distributed light through the playing area, can improve both spectators’ experience and players’ performance.^86^ According to the related guidelines,^17,87^ requirements for lighting in sports facilities are relatively stricter than for other public buildings, because the high speed of action requires superior visual quality to locate a moving object precisely.^88^ Recently, Shi et al.^89^ defined several new luminance parameter thresholds for users’ visual comfort in a gymnasium by considering subjective responses.

Lighting conditions in sports facilities can also have non-visual (such as psychological and physiological) effects on occupants,^90,91^ and might induce violent reactions from spectators. According to Amorim et al.,^63^ a dynamic LED (light-emitting diode) illumination is a good way to decrease violence among spectators. They suggested changing the illumination levels and colour temperature of the LED illuminance in spectators’ areas at different time periods (before, during and after the match), so that spectators can better enjoy the match.

More importantly, light has been found to be an effective microbiocidal agent since the 19^th^ century. More than 100 years ago, Downes and Blunt^92^ observed that light is inimical to bacteria reproduction and development, and direct insolation could destroy the germs in solutions. Since the outbreak of COVID-19, many light-based technologies have been studied and suggested to be used to inactivate SARS-CoV-2.^93^ For example, Kumar et al.^94^ proposed a design with a Xenon lamp source and aluminium high reflecting surface to disinfect the virus attached to the surfaces of phones, keys, clothes, etc. Fischer et al.^95^ compared several decontamination methods (including UV (ultraviolet) radiation, 70°C dry heat, 70% ethanol and vaporized hydrogen peroxide) and found that UV light could rapidly inactive SARS-CoV-2 from the steel surface. Sabino et al.^93^ reviewed several light-based strategies (such as ultraviolet germicidal irradiation (UVGI), antimicrobial blue light and ultrafast laser irradiation at low irradiance), and summarized advantages and disadvantages of their possible applications. UVGI was claimed to be an effective method that can be used in both air and water disinfection.

Most UV-C systems make use of UV-C with a wavelength of 254 nm, which is harmful to skin and eyes and therefore, should not be used in the vicinity of people. UV-C with a wavelength of 222 nm, on the other hand, is not harmful to people, and has shown to be effective in inactivating, for example, SARS (severe acute respiratory syndrome) and MERS (Middle East respiratory syndrome),^96,97^ but is as far as is known not yet available on the market. What is important to note, is that UV-lights can only deactivate the pathogens that they can ‘see’.

UV-C cleaning is used in ‘in-duct’ applications, combined with the air conditioning system, for example, for operating theatres where recirculation is a must.^98^ Upper-room ultraviolet light applications, in which lamps are placed in the upper part of the room, either on the walls or mounted on the ceiling, directing the UV-C light into the upper zone with louvres and limiting UV-exposure in the occupied space, have been considered for use in crowded, poorly ventilated environments.^99^ Also, small devices and robots are available to disinfect products and even surfaces of entire spaces with UV-C light.

Apart from the effect on occupants, lighting systems can also play an important role in energy consumption. Suresh et al.^100^ and Salis et al.^86^ conducted an on-site investigation on lighting quality and energy consumption in a table tennis court and a squash court, respectively, and found that the lighting system in neither of these two sports courts was energy efficient. Therefore, they proposed a new lighting design by using less but uniformly placed energy-efficient LED luminaires. Nowadays, the usage of LED light is very common in the Netherlands because of the ‘transition to LED lighting’ measure advocated by the Dutch governments,^101,102^ however, the high costs are still a problem. Therefore, several Dutch scientists proposed a new technology that combines LED lighting with a Direct Current (DC) power system to improve both performance and costs of LED lights.^103^

#### Interactions between the IEQ factors

Interactions can occur between the different IEQ factors (air quality, thermal quality, acoustical quality and visual quality). Table 6 presents a list of possible interactions between the different IEQ factors. Unfortunately, few studies including interactions have been undertaken in sports facilities.

**Table 6.** Interactions between the different IEQ factors.

Factor	Action	Factor	Interaction
Thermal quality	Increase of temperature	Indoor air	Increase emissions
	Use of solar screens to prevent overheating	Lighting	Reduction of daylight entrance
Visual quality	Use of internal glass walls	Acoustical	Possible sound reflections
	Increase glass surface area	Thermal	Possible overheating
Acoustical quality	Sound adsorption material in air supply ducts	Indoor air	Possible decrease supply air quality
	Closing windows	Indoor air	Decrease outdoor air
Indoor air quality	Increase ventilation rate/opening windows	Thermal Acoustical	Possible draught Possible noise pollution
	Increase rel. humidity	Thermal	Influence on mould growth
	Removal fleecy materials	Acoustical	Reduction sound absorption

Most studies on interactions have been undertaken in schools or offices, and between IAQ and thermal quality.^104–106^ For example, Chatzidiakou et al.^107^ developed a model to demonstrate the relationship between indoor air temperature and CO_2_ concentration. They found that a high CO_2_ concentration (e.g. above 1500 ppm) in a normal classroom with 30 occupants might be a sign of overheating (e.g. above 25°C). In naturally ventilated spaces, opening windows is the only way to guarantee enough ventilation and keep a good IAQ, but at the same time this behaviour might influence occupants’ thermal sensations or even threaten their thermal comfort, especially during wintertime.^108^ In mechanically ventilated spaces, increasing the ventilation rate is the common way to improve IAQ, however, this can cause draughts and influence occupants’ thermal sensations.^109^

Another example of interactions is the use of mobile HEPA (high-efficiency particulate air) filter systems, which have been shown to clean the air of particles around the size of SARS-CoV-2. Tests with a mobile HEPA filter system in a semi-laboratory environment showed that the HEPA filter’s cleaning performance depends on the setting of the system and the position in a room.^110^ Moreover, the system can result in unacceptable noise levels and draught effects, and more than one system is needed in one classroom, resulting in even higher noise and draught effects. In Germany, requirements for mobile air purifiers to reduce airborne transmission of infectious diseases have been developed, including also aspects such as noise generation and comfort aspects.^111^

In terms of the interaction between IAQ and acoustical quality, in most cases, they appear to be irreconcilable. In mechanical ventilated spaces, a positive correlation between ventilation rate and the noise level was found by Khaleghi et al.^112^: the higher the ventilation rate, the higher the noise level caused by the HVAC system. In another study,^113^ for naturally ventilated spaces was observed, that when the windows are closed, acoustical quality was acceptable but the IAQ was not because of high particle concentrations and low ventilation rates. When the windows were open, IAQ improved but the noise level exceeded the required level. Additionally, some acoustical treatments, such as acoustical ceilings or carpets, might also cause interaction effects between acoustical quality and air quality, because these materials can emit VOCs (volatile organic compounds) and shed fibres.^114^ Although acoustical treatment can improve acoustical quality by reducing the RT, a lower IAQ could be induced.^112^

## Discussion

### Main indicators to assess IEQ in sports facilities

#### Indoor air quality

The main indicators used to assess IAQ in sports facilities are dose-related indicators, which can vary per type of facility. Sports halls, specifically larger halls where different team activities and disciplines can take place (from volleyball to rock climbing, etc.) are the most studied type of sports facility, and hence a wider range of pollutants has been investigated. The main pollutants investigated in sports halls are NO_x_, magnesia alba, acetone, formaldehyde, PM_10_ and VOCs. Fitness centres, defined in this study as smaller spaces in which smaller groups of workouts and fitness routines can be exercised, tend to be investigated less than sports halls, and the main pollutants investigated were PM_10_, VOCs, fungi and yeasts, and bacteria on surfaces and in air. Finally, swimming pools, because of the special cleaning treatment required and the high humidity present, these have been investigated with a different scope of pollutants, with the most common ones being PM_2.5_, VOCs, fungi and yeasts, and different compounds resulting from bleaching processes.

While in France, Belgium and Germany national schemes for emissions of construction products have been introduced, the indoor air quality guidelines of the WHO^13^ as well as the different available labelling schemes for building materials are not used in the Netherlands.^20^ In the Netherlands, instead of pollutant concentrations, the main focus of IAQ assessment is on ventilation performance. As shown in Table 4, the minimum ventilation rates are different in different sports facilities or different areas; and these values are different for different seasons.^36^ In some guidelines, two different values with different units (l/s/p or l/s/m^2^) are given and can be selected based on the pollutant sources present. In most sports facilities, occupants are the main air pollutant sources, therefore l/s/p has been suggested to be used.

#### Ventilation and CO_2_

Current guidelines for air quality indoors are mainly based on the CO_2_ concentration in air that is allowed.^16,21^ CO_2_ is used as an indicator for the presence of people. With every breath we take, we exhale CO_2_. The amount of air ventilated, in the case of mechanical ventilation, is usually assessed by air velocity sensors in the supply duct and/or exhaust duct.

Whether CO_2_ is a good indicator for exhaled ‘infectious’ aerosols, is not known yet. CO_2_ is a gas, and exhaled aerosols and droplets are no gasses, and most likely do not all behave as gasses. The CO_2_ levels set are based on ‘comfort’, not on health. Moreover, in a study at secondary schools, the CO_2_ concentration in a classroom was found to differ per location depending on the ventilation regime, the dimensions of the room and occupancy.^115^ Also, the outdoor CO_2_ concentration can differ during the day and per location.

To cope with the risk of airborne transmission, the most used model has been the Wells-Riley model.^116^ Based on this model, it is theoretically possible to calculate the infection risk for a certain ventilation rate and the number of persons present, assuming one infected person. Unfortunately, this model or equation has a number of limitations: (a) it does not account for differences between persons; (b) a mixing situation is assumed (the concentration of infectious aerosols is homogeneous in indoor spaces); and (c) this uniform concentration is assumed over time to be constant, and therefore the ongoing inhalation of the concentration is constant over time.^7^

#### Other IEQ factors

For the other IEQ factors (thermal, acoustical and lighting quality), the indicators used in standards and guidelines are mainly dose-related indicators. For some of the parameters, such as air velocity and horizontal illuminance, the requirements are specific and clear, while for others, such as relative humidity and reverberation time, the requirements are either not mentioned or not consistent in different standards, which makes it difficult to assess the performance of these indicators in various sports facilities.

For those parameters that have clear requirements and can be easily measured (such as air temperature), even if the measured results meet the requirements, it does not mean that all occupants in the sports facilities are comfortable. Kosonen et al.^117^ found that occupants’ perceived IEQ was, in many cases, lower than the prescribed standards level. For sports facilities, the following reasons might cause this mismatching between occupants’ perceived IEQ and the physically measured IEQ. First, people in different areas of the sports facilities (e.g. audience area and activity area) have different requirements^70^; second, athletes or players in the same area might also have different IEQ perceptions because of different exercise intensity, body mass index (BMI), age or gender^118^; third, all the IEQ factors might interact with each other, so even if the parameters themselves meet the requirements, the interactions between them might cause discomfort of occupants.^119^

### Building-related and occupant-related indicators

Dose-response related threshold levels or ranges of levels are usually set for an average person, not accounting for differences between people, situations and interactions between the different indicators, both at human and environmental levels. Both occupant-related and building-related indicators are important to account for.^120^

#### Building-related indicators

With regards to building-related indicators, White et al.^121^ observed extensive mould and dampness (which could deteriorate IAQ) in two prefabricated buildings in Australia and their investigation indicated that these problems were caused by flaws in building design and construction. Kim and De Dear^122^ found that building layout and the amount of space (which are easily ignored in previous IEQ research) also could influence occupants’ overall satisfaction with IEQ. Similarly, Wang and Zamri^123^ also identified space layout as one of the important factors that have significant relationships with occupants’ IEQ satisfaction. Although these correlations between building layout/construction materials and IEQ in sports facilities have been rarely studied before, the principle behind these relationships should be the same. Therefore, floor area, layout and covering materials in sports facilities can also affect the IEQ in these buildings.

The conclusion drawn from previous studies is that building-related indicators are needed in addition to dose-related indicators prescribed in most standards and guidelines. Some requirements for building-related indicators have been set (see Table 2), but those are in general (except for the colours reflection factor of floor, walls and ceiling materials) not meant to improve the IEQ. In Table 7 a list of building-related indicators is presented that would be worthy to include and have been suggested in previous studies.^124,125^ More research is required to better define the criteria/requirements for these indicators for different sports facilities, occupants, activities and spaces. In particular is the minimum required height of the space, such as a sports hall, with regards to the ventilation regime, the location of the air supplies and exhausts, the direction, temperature and velocity of the flows (determining the distribution) and resulting ventilation efficiency, local and overall.

**Table 7.** Suggested building-related indicators for sports facilities.

Component/topic	Building-related indicator
Ventilation regime	Ventilation type (local) ventilation efficiency airflow pattern
Natural ventilation	Windows location and dimensions passive grills
Mechanical ventilation	Location of air supply and exhausts grilles direction flow maintenance schedule
Air cleaning	Type of air filter air cleaning devices
Floor	Type of wall material mission label colour reflection factor hard/fleecy material
Walls	Type of ceiling material emission label colour reflection factor
Ceiling	Type of ceiling material emission label height colour reflection factor
Cleaning	Cleaning schedule Cleaning products
Windows	Window frame colour versus wall colour single/double/triple glazing
Lighting	Type of lighting natural or artificial reflection on the surface
Sound absorption material	Presence of sound absorption material location of sound absorption material
Heating and cooling system	Type of heating system location of radiators (if there are) type of cooling system

#### Occupant-related indicators

Several air pollutants are known to cause a variety of detrimental health effects, depending on the exposure time and concentrations of pollutants. However, in the case of halls and fitness centres, due to the increased physical activity of occupants exercising, and hence, due to their increased respiration rate, these persons might be at a greater risk than spectators or personnel. This discrepancy with different occupants is also true for other IEQ factors in sports facilities, for example, sportspeople in the playing area have more strict requirements on thermal quality since it can influence their performance, while for the audience area, wide ranges of requirements are applied, as long as the thermal comfort of the audience is met.^73,126^ Indeed, IEQ standards and guidelines for various sports facilities have not been customized, and do not take into account the fact that different humans, depending on their physiology, activity or unique reactions, can have different health effects even when exposed to the same indoor environment.

In Table 8, a list of occupant-related indicators is presented that would be worthy to include and have been suggested in previous studies.^127,128^

**Table 8.** Suggested occupant-related indicators for sports facilities.

Component/topic	Occupant-related indicator
Personal characteristics	Number of occupants
	age range
	sex
	duration of stay
	activity level
IEQ and health	Needs to deal with diseases and disorders, such as: allergies, asthma, diabetes, etc.
IEQ and comfort	Preferences for IEQ to perform well, such as preferences for light (brighter/darker), background noise (non/a lot), temperature (warm/cold), draft/still air, etc.

### Ventilation and SARS-COV-2

To decrease the risk of far-range airborne transmission of SARS-CoV-2, the use of ‘proper’ ventilation measures has been recommended.^4,129–132^ ‘Proper’ ventilation means to provide sufficient, and effective ventilation that ensures the supply of ‘clean’ air and exhausts polluted (‘infected’) air from the breathing zones of each individual person, without passing through the breathing zones of other persons, and preferably without recirculation of air. Air cleaning or/and disinfection devices can be added, if general ventilation seems not enough or recirculation (re-use of air) cannot be avoided.^133^

To remove pollutants, several types of ventilation systems are applied in sports facilities: (a) natural ventilation, established by just opening a window, or (b) mechanical ventilation varying from just an exhaust vent to very advanced air conditioning systems that supply and exhaust the air.^134^ While natural ventilation is an uncontrolled form, mechanical ventilation gives the possibility to control the amount of air that is supplied, exhausted and/or re-used. Natural ventilation depends on several environmental aspects such as the wind and temperature outdoors, but also on the dimensions of the space and the possibility of having openings in the façade. In addition to the ventilation type, also different ventilation principles can be selected such as mixing ventilation, displacement ventilation, cross ventilation and personal ventilation. The most commonly applied mechanical ventilation principle in sports facilities is mixing ventilation, in which air is supplied up (near or via the ceiling) with an air velocity high enough to realize mixing in the space, and therefore the number of ‘infectious’ aerosols in the air is reduced, but not all of them are removed. Displacement and cross ventilation move the air horizontally or vertically through a space, ideally replacing polluted air with ‘fresh’ air. With displacement ventilation air is supplied low nearby or via the floor with a low velocity and exhausted up in the space. Personal ventilation supplies and/or exhausts air in the breathing zone of each individual person.

It is clear that ventilating a space reduces the concentration of possible ‘infectious’ aerosols. How those ‘infectious’ aerosols distribute and possibly meet a healthy person on the way who can inhale them, is difficult to predict with ventilation at room level. To really reduce the risk of transmission to nearly zero, the possible ‘infectious’ aerosols should be extracted as close as possible to the source. Personal ventilation is a possibility when the directions of airflows are considered. Most available personal ventilation systems are focused on the supply of fresh air into the breathing zone, not on the exhaust.^135^ This type of personal ventilation is, for example, used in fitness centres’ treadmills, but may not be functional in a sports hall. Moreover, the consideration should not only be about how much fresh air is supplied but also about where it is ventilated and distributed through the space, in relation to the activities taking place and the occupancy over time.^7^

## Conclusions and recommendations

The outcome of this literature study makes several contributions to the current literature.

First, it shows that current standards and guidelines for IEQ in sports facilities are mainly focused on the dose-related indicators such as concentration of air pollutants, temperature level, relative humidity and ventilation rate in sports facilities. Occupant-related indicators (such as age, diseases and disorders, preferences and needs, and activity level), and building-related factors (such as certain measures or characteristics of a building and its components) can also play an important role in occupants’ health and comfort, and should therefore be considered. The study recommends the identification of preferences and needs (profiles) of different occupants for different sports facilities and activities, as well as building-related indicators to realize the required environmental conditions (dose-related indicators) for each profile.^136^

Second, in the studies presented in this review little consideration is given to the fact that ventilation may play an important role in the air quality of the location. Further studies and the design of buildings should consider those different ventilation strategies that may be more appropriate for specific types of locations within sports facilities, based on the activities taking place or building and furnishing materials used. It is recommended to consider both occupant and building-related indicators in future standards and studies on IEQ in sports facilities.

Third, few investigations have been performed on the transmission of SARS-CoV-2 in sports facilities. From studies in other settings (e.g. educational settings, restaurants and care homes) with a high density of people, clearly, the question should not only be about which ventilation rates are required to protect against infection transmission, but also how the space should be ventilated for a specific situation. The recommendation is to apply ventilation that not only focuses on the ventilation of a space but also provides a range of ventilation options that fulfil the demands of the occupants over time, whether related to health or comfort.^7^ The applied ventilation strategies should not just focus on the amount of ventilation required for a space, but also on the how (where), and when the ventilation is needed.

Finally, most previous studies, standards and guidelines only focus on one IEQ factor, especially on IAQ. However, much uncertainty still exists about the relationship between IEQ factors. Measures to improve IAQ could have an effect on other factors and consequently affect the health and comfort of the occupants. Therefore, cross-modal effects between the IEQ factors are suggested to be considered for future standards development.
